# Efficacy and safety of erenumab in Japanese migraine patients with prior preventive treatment failure or concomitant preventive treatment: subgroup analyses of a phase 3, randomized trial

**DOI:** 10.1186/s10194-021-01313-8

**Published:** 2021-09-18

**Authors:** Koichi Hirata, Fumihiko Sakai, Takao Takeshima, Noboru Imai, Yasuhiko Matsumori, Ryuji Yoshida, Yotaro Numachi, Cheng Peng, Daniel D. Mikol, Sunfa Cheng

**Affiliations:** 1 Department of Neurology Headache Center, Dokkyo Medical University, 880 Kitakobayashi, Mibu, Shimotsuga District, Tochigi, 321-0293 Japan; 2 Saitama International Headache Center, Saitama, Japan; 3 Department of Neurology Headache Center, Tominaga Hospital, Osaka, Japan; 4 Department of Neurology, Japanese Red Cross Shizuoka Hospital, Shizuoka, Japan; 5 Sendai Headache and Neurology Clinic, Sendai, Japan; 6 Amgen K.K, Tokyo, Japan; 7 Global Biostatistical Science, Amgen Inc., Thousand Oaks, CA USA; 8 Global Development, Amgen Inc., Thousand Oaks, CA USA

**Keywords:** Concomitant preventive treatment, Erenumab, Migraine, Prior preventive treatment failure

## Abstract

**Background:**

These subgroup analyses of a Phase 3, randomized, double-blind, placebo-controlled study evaluated the efficacy and safety of erenumab 70 mg in Japanese migraine patients with/without prior preventive treatment failure(s) (“failed-yes” and “failed-no” subgroups) and with/without concomitant preventive treatment (“concomitant preventive-yes” and “concomitant preventive-no” subgroups).

**Methods:**

Overall, 261 patients were randomized; 130 and 131 patients to erenumab 70 mg and placebo, respectively. Subgroup analyses evaluated the change from baseline to Months 4–6 in mean monthly migraine days (MMD) (primary endpoint), achievement of a ≥50% reduction in mean MMD, and change from baseline in mean monthly acute migraine-specific medication (MSM) treatment days. Treatment-emergent adverse events were also evaluated.

**Results:**

Of the 261 patients randomized, 117 (44.8%) and 92 (35.3%) patients were in the failed-yes and concomitant preventive-yes subgroups, respectively. Erenumab 70 mg demonstrated consistent efficacy across all subgroups, with greater reductions from baseline in mean MMD versus placebo at Months 4–6 (treatment difference versus placebo [95% CI], failed-yes: − 1.9 [− 3.3, − 0.4]; failed-no: − 1.4 [− 2.6, − 0.3]; concomitant preventive-yes: − 1.7 [− 3.3, 0.0]; concomitant preventive-no: − 1.6 [− 2.6, − 0.5]). Similar results were seen for achievement of ≥50% reduction in mean MMD and change from baseline in mean monthly acute MSM treatment days. The safety profile of erenumab 70 mg was similar across subgroups, and similar to placebo in each subgroup.

**Conclusion:**

Erenumab was associated with clinically relevant improvements in all efficacy endpoints and was well tolerated across all subgroups of Japanese migraine patients with/without prior preventive treatment failure(s) and with/without concomitant preventive treatment.

**Trial registration:**

Clinicaltrials.gov. NCT03812224. Registered January 23, 2019.

**Supplementary Information:**

The online version contains supplementary material available at 10.1186/s10194-021-01313-8.

## Introduction

Migraine, i.e. episodic migraine (EM) and chronic migraine (CM), is a debilitating headache disorder that significantly impairs functioning and quality of life [1–3], with an annual prevalence of 8.4% in Japan [4]. The goal of preventive treatment is to reduce the frequency and severity of migraine; however, unmet needs for new treatments remain for Japanese patients with migraine due to low utilization rates, insufficient efficacy, and/or poor tolerability of existing preventive therapies [5–9].

Erenumab (erenumab-aooe in the United States) is a fully human monoclonal antibody that selectively targets and blocks the calcitonin gene-related peptide receptor [10–13]. Previously, a double-blind, placebo-controlled, Phase 2 study of Japanese patients with EM demonstrated significantly greater reductions with erenumab 70 mg and 140 mg once monthly versus placebo in mean monthly migraine days (MMD) and mean monthly acute migraine-specific medication (MSM) treatment days, along with significantly greater odds of achieving a ≥50% reduction from baseline in mean MMD [14]. In Phase 2 and 3 studies outside of Japan, the efficacy of erenumab has been demonstrated in patients with EM or CM who have previously failed migraine preventive treatment [15–17]. However, the efficacy of erenumab among Japanese patients who have previously failed preventive treatment or who are currently receiving a standard of care preventive treatment remains to be elucidated.

Recently, a Phase 3, randomized, double-blind, placebo-controlled study assessed the efficacy and safety of erenumab 70 mg for the prevention of migraine in 261 Japanese patients with EM or CM [18]. Here, we report the results of subgroup analyses of the main Phase 3 study [18] that evaluated the efficacy and safety of erenumab 70 mg treatment in the subgroups of Japanese patients with/without prior preventive treatment failure(s) and with/without concomitant preventive treatment.

## Methods

### Study design and patients

These were subgroup analyses of data from a Phase 3, randomized, double-blind, placebo-controlled study of 261 Japanese patients with EM or CM (ClinicalTrials.gov identifier: NCT03812224) [18]. The study comprised an initial screening phase (up to 3 weeks), a 4-week baseline phase, and a 24-week double-blind treatment phase (DBTP), followed by a 28-week open-label extension period and an 8-week follow-up period (Supplemental Fig. 1). In the DBTP, patients were randomized (1:1) to receive erenumab 70 mg or placebo once monthly subcutaneously for 24 weeks. This study reports efficacy and safety data from the 24-week DBTP only.

The study enrolled Japanese patients with EM or CM aged 20–65 years, with a history of migraine (International Classification of Headache Disorders, 3rd edition criteria) with or without aura for ≥12 months before screening. CM was defined as ≥15 headache days per month, of which ≥8 headache days on average across the 3 months prior to screening met the criteria for migraine days. EM was defined as <15 headache days per month, of which ≥4 headache days on average across the 3 months prior to screening met the criteria for migraine days. During the baseline phase, patients were required to have the same migraine type as prior to screening and demonstrate ≥80% compliance with a daily electronic diary (eDiary) used to record migraine symptoms.

The main exclusion criteria included age >50 years at migraine onset, body mass index >40 kg/m^2^, history of cluster/hemiplegic migraine, migraine with continuous pain, no therapeutic response to ≥3 prior migraine preventive classes (defined as no reduction in headache frequency, duration, or severity after 6 weeks of treatment at the generally accepted therapeutic dose, as judged by the investigator), or any significant medical condition that precluded study entry.

### Subgroups

Patient subgroups were defined on the basis of prior and concomitant migraine preventive treatment status. The “failed-yes” subgroup included patients who had previously failed ≥1 prior preventive treatment category due to lack of efficacy and/or unacceptable tolerability, as recorded by the investigator. The “failed-no” subgroup included patients who had never failed prior preventive treatment (composed of patients who had not previously received preventive treatment or received preventive treatment and had not failed). The “concomitant preventive-yes” subgroup included patients receiving a concomitant standard of care migraine preventive treatment and the “concomitant preventive-no” subgroup included patients not receiving a concomitant migraine preventive treatment.

Migraine preventive treatments were defined according to the American Academy of Neurology/American Headache Society [19], and Japanese Headache Society guidelines [6]. The following were the categories of prior migraine preventive treatments: topiramate; beta-blockers (e.g. propranolol or metoprolol); tricyclic antidepressants (e.g. amitriptyline or nortriptyline); divalproex sodium or sodium valproate; calcium channel blockers (e.g. flunarizine, verapamil, lomerizine); serotonin-norepinephrine reuptake inhibitors (e.g. venlafaxine, desvenlafaxine, duloxetine, and milnacipran); botulinum toxin; antihypertensives (lisinopril or candesartan); or other medications. Except for the category of “other medications,” any of the aforementioned medications were considered a concomitant migraine preventive medication, along with clonidine, guanfacine, cyproheptadine, pizotifen, methysergide, butterbur, feverfew, magnesium, and riboflavin.

### Assessments and endpoints

Headache data were captured daily via eDiary measurement during the baseline period and the DBTP. The primary efficacy endpoint was the change from baseline in mean MMD. A migraine day was defined as any calendar day on which the patient had onset, continuation, or recurrence of a qualified migraine (i.e. migraine with or without aura lasting for ≥4 h with either ≥2 pain features or ≥1 associated nonpain symptom, or both) as recorded in the eDiary. Any calendar day on which acute MSM was used was also counted as a migraine day regardless of the headache duration, pain features, and associated symptoms. Secondary efficacy endpoints were the proportion of patients achieving a ≥50% reduction from baseline in mean MMD and the change from baseline in mean monthly acute MSM treatment days.

All efficacy endpoints were assessed over the last 3 months (Months 4–6) of the DBTP. Treatment-emergent adverse events (TEAEs) were collected throughout the DBTP and graded according to Common Terminology Criteria for Adverse Events (CTCAE) version 4.03.

### Statistical analysis

All statistical analyses were conducted using SAS version 9.2 (SAS Institute Inc., Cary, NC, USA). All efficacy analyses in the prior treatment failure subgroups were prespecified, and efficacy analyses in the concomitant preventive treatment subgroups were post hoc. The full analysis set included all randomized patients. The efficacy analysis set included all patients who received ≥1 dose of erenumab 70 mg or placebo and had ≥1 change from baseline measurement in MMD during the DBTP. The safety analysis set included all randomized patients who received ≥1 dose of erenumab 70 mg or placebo, unless a patient received the incorrect dose during the DBTP. Changes from baseline in mean MMD and mean monthly acute MSM treatment days over Months 4–6 were analyzed using generalized linear mixed-effect models, which included treatment, visit, treatment-by-visit interaction, stratification factor of migraine type (EM or CM), and baseline value as covariates and assumed a first-order autoregressive covariance structure. An additional stratification factor of migraine preventive treatment status (ever used or never used) was not included in the adjusted models. Adjusted analysis results were obtained using contrasts. The least squares mean (LSM) changes from baseline with 95% confidence intervals (95% CIs) for each treatment group, the treatment difference (erenumab 70 mg − placebo) with the 95% CI, and nominal *p*-values without multiplicity adjustment were calculated. Achievement of a ≥50% reduction from baseline in mean MMD was analyzed using a stratified Cochran-Mantel-Haenszel test (CMH), with missing data imputed as nonresponse. Adjusted odds ratios (ORs) and associated *p*-values were obtained from the CMH test, stratified by migraine type (EM or CM). Treatment-by-subgroup interaction *p-*values were obtained from generalized linear mixed models adjusting for treatment group, stratification factor of migraine type (EM or CM), subgroup and treatment-by-subgroup interaction, with data imputed using the nonresponder imputation method.

## Results

### Baseline characteristics

A total of 261 patients were randomized: 130 patients to the erenumab 70 mg group and 131 patients to the placebo group. Of the 261 patients randomized, 117 (44.8%) patients were in the failed-yes subgroup. The 144 patients in the failed-no subgroup included 59 (41.0%) treatment-naïve patients, while 85 (59.0%) patients had prior preventive treatment use but had not failed. There were 92 (35.2%) patients in the concomitant preventive-yes subgroup (Table 1). There was a higher proportion of patients with ≥1 prior preventive treatment failure(s) in the concomitant preventive-yes subgroup compared with the concomitant preventive-no subgroup: 50 (54.3%) versus 67 (39.6%) patients, respectively. Calcium channel blockers were the most common concomitant preventive treatment received by patients in the concomitant preventive-yes subgroup (Supplemental Table 1).

**Table 1:** Baseline characteristics

	Patient subgroups							
	Prior preventive treatment failure				Concomitant preventive			
	Yes (*N* = 117)		No (*N* = 144)		Yes (*N* = 92)		No (*N* = 169)	
	Erenumab 70 mg (*N* = 59)	Placebo (*N* = 58)	Erenumab 70 mg (*N* = 71)	Placebo (*N* = 73)	Erenumab 70 mg (*N* = 40)	Placebo (*N* = 52)	Erenumab 70 mg (*N* = 90)	Placebo (*N* = 79)
Sex, female, n (%)	54 (91.5)	52 (89.7)	57 (80.3)	64 (87.7)	35 (87.5)	48 (92.3)	76 (84.4)	68 (86.1)
Age, years, mean (SD)	44.8 (9.5)	44.9 (8.9)	43.6 (7.7)	44.3 (9.7)	44.4 (9.4)	44.6 (9.0)	44.0 (8.2)	44.5 (9.6)
Migraine type, n (%)								
CM	30 (50.8)	31 (53.4)	22 (31.0)	19 (26.0)	18 (45.0)	24 (46.2)	34 (37.8)	26 (32.9)
EM	29 (49.2)	27 (46.6)	49 (69.0)	54 (74.0)	22 (55.0)	28 (53.8)	56 (62.2)	53 (67.1)
Disease duration, years, mean (SD)	19.4 (11.2)	18.4 (10.5)	16.3 (11.6)	19.3 (13.1)	20.7 (12.6)	19.0 (12.2)	16.4 (10.8)	18.8 (11.9)
MMD, mean (SD)	13.8 (6.0)	13.9 (6.0)	11.2 (5.8)	10.2 (4.9)	12.6 (5.9)	13.0 (6.2)	12.3 (6.1)	11.1 (5.3)
Monthly headache days, mean (SD)	15.4 (6.6)	16.1 (6.5)	12.8 (6.3)	11.7 (5.6)	14.4 (6.1)	14.9 (6.9)	13.9 (6.7)	12.8 (5.9)
Any acute MSM use, n (%)	57 (96.6)	55 (94.8)	68 (95.8)	69 (94.5)	39 (97.5)	48 (92.3)	86 (95.6)	76 (96.2)
Monthly acute MSM treatment days, mean (SD)	10.6 (6.4)	10.4 (5.5)	8.7 (5.3)	8.3 (4.8)	9.2 (5.7)	9.2 (5.4)	9.7 (6.0)	9.2 (5.2)
Prior preventive treatment failure status, n (%)								
Never failed (including nonusers)	0 (0.0)	0 (0.0)	71 (100.0)	73 (100.0)	18 (45.0)	24 (46.2)	53 (58.9)	49 (62.0)
Failed ≥1 prior preventive class	59 (100.0)	58 (100.0)	0 (0.0)	0 (0.0)	22 (55.0)	28 (53.8)	37 (41.1)	30 (38.0)
Failed ≥2 prior preventive classes	30 (50.8)	33 (56.9)	0 (0.0)	0 (0.0)	12 (30.0)	15 (28.8)	18 (20.0)	18 (22.8)

Data are for the full analysis set. *CM* chronic migraine, *EM* episodic migraine, *MMD* monthly migraine days, *MSM* migraine-specific medication, *SD* standard deviation

Across the subgroups, the mean age was 44–45 years and most patients were female. The mean (standard deviation [SD]) number of MMD at baseline ranged from 10.2 (4.9) to 13.9 (6.0), and the mean (SD) number of monthly acute MSM treatment days ranged from 8.3 (4.8) to 10.6 (6.4) (Table 1). The failed-yes and concomitant preventive-yes subgroups generally had more severe disease than the failed-no and concomitant preventive-no subgroups, based on the greater proportion of patients with CM and the higher number of mean MMD. The concomitant preventive-yes subgroup had more patients who had previously failed prior preventive treatment than the concomitant preventive-no subgroup. Patients in the failed-yes subgroup had a higher number of mean monthly acute MSM treatment days than those in the failed-no subgroup (Table 1).

### Change from baseline in mean MMD

**Fig. 1:**
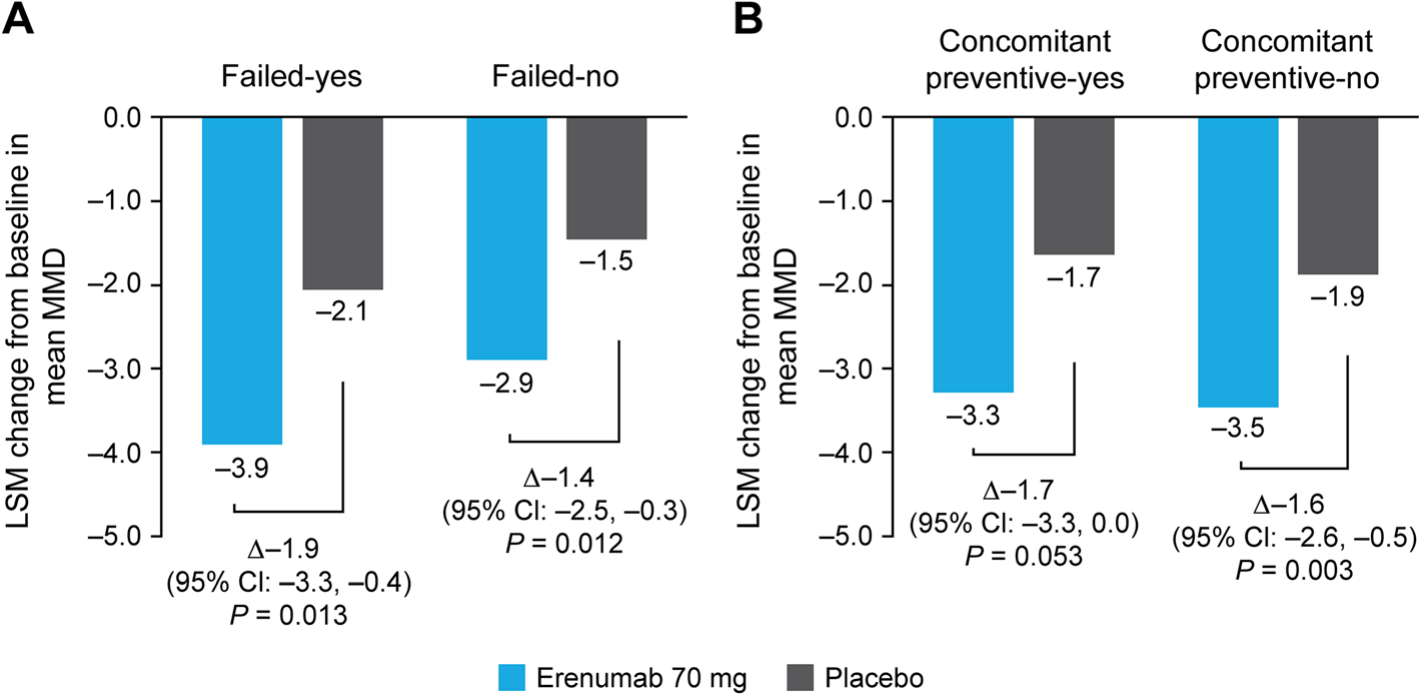
LSM change from baseline in mean MMD in (**A**) failed-yes/no and (**B**) concomitant preventive-yes/no subgroups. *CI* confidence interval, *LSM* least squares mean, *MMD* monthly migraine days

From baseline to Months 4–6, significant reductions in mean MMD occurred with erenumab 70 mg versus placebo in all patient subgroups. The differences in LSM change from baseline in mean MMD with erenumab 70 mg versus placebo were − 1.9 (95% CI: − 3.3, − 0.4; *P* = 0.013) and − 1.4 (95% CI: − 2.5, − 0.3; *P* = 0.012) in the failed-yes and failed-no subgroups, respectively. The differences in LSM change from baseline in mean MMD with erenumab 70 mg versus placebo were − 1.7 (95% CI: − 3.3, 0.0; *P* = 0.053) and − 1.6 (95% CI: − 2.6, − 0.5; *P* = 0.003) in the concomitant preventive-yes and concomitant preventive-no subgroups, respectively (Fig. 1).

*P*-values for treatment-by-subgroup interactions were 0.70 and 0.93 for the failed-yes/no and concomitant preventive-yes/no subgroups, respectively, confirming that the efficacy of erenumab 70 mg was similar regardless of whether a patient failed or did not fail a prior preventive treatment or was receiving or not receiving a concomitant treatment.

### ≥50% reduction from baseline in mean MMD

**Fig. 2:**
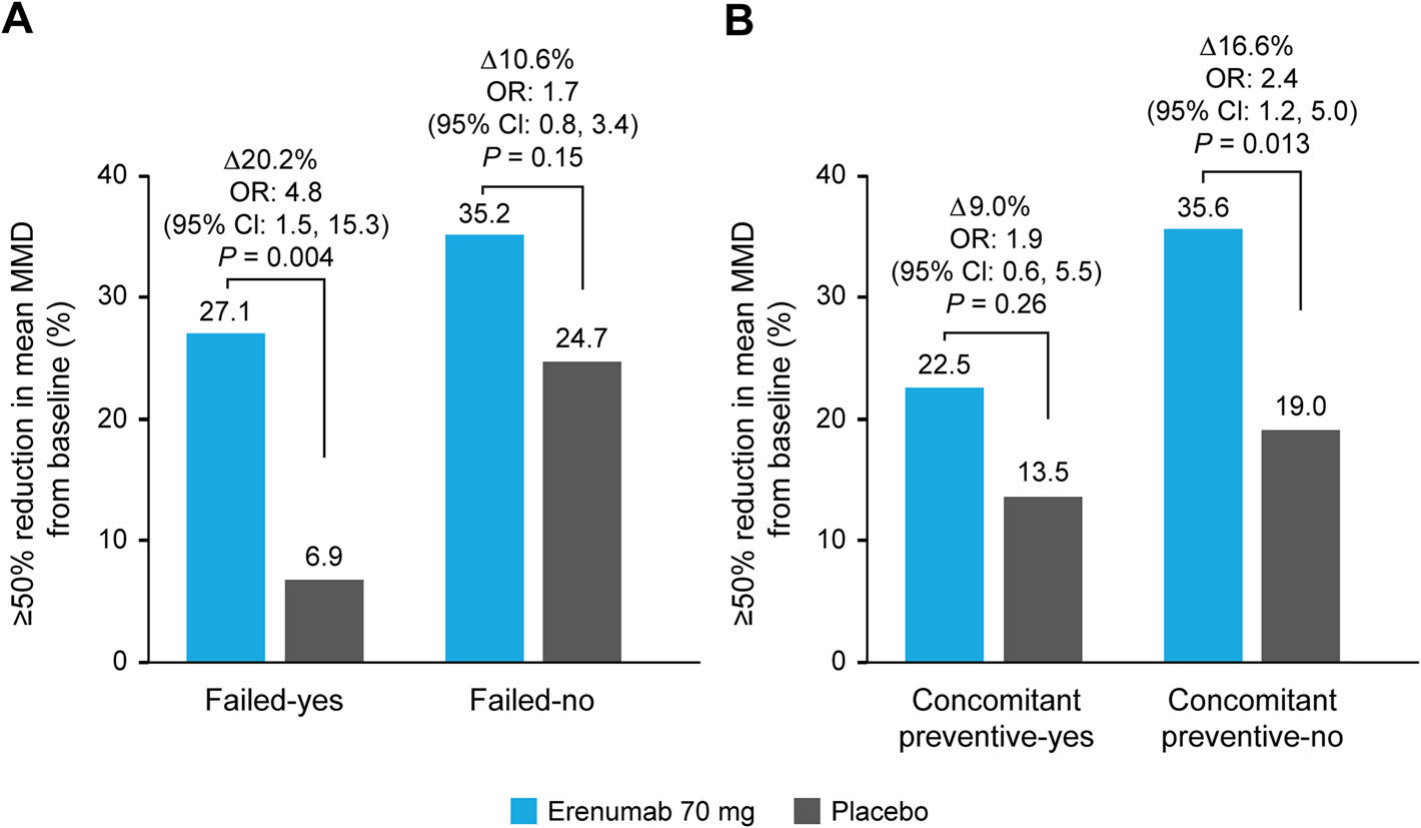
≥50% reduction in mean MMD from baseline in (**A**) failed-yes/no and (**B**) concomitant preventive-yes/no subgroups. *CI* confidence interval, *MMD* monthly migraine days, *OR* odds ratio

At Months 4–6, a higher proportion of patients treated with erenumab 70 mg achieved a ≥50% reduction in mean MMD versus placebo in all patient subgroups (Fig. 2). In patients treated with erenumab 70 mg, a ≥50% reduction in mean MMD was achieved by 27.1%, 35.2%, 22.5%, and 35.6% of patients in the failed-yes, failed-no, concomitant preventive-yes, and concomitant preventive-no subgroups, respectively.

The common ORs versus placebo of achieving a ≥50% reduction in mean MMD were 4.8 (95% CI: 1.5, 15.3; *P* = 0.004) and 1.7 (95% CI: 0.8, 3.4; *P* = 0.15) in the failed-yes and failed-no subgroups, respectively, and 1.9 (95% CI: 0.6, 5.5; *P* = 0.26) and 2.4 (95% CI: 1.2, 5.0; *P* = 0.013) in the concomitant preventive-yes and concomitant preventive-no subgroups, respectively (Fig. 2).

*P*-values for treatment-by-subgroup interactions were 0.12 and 0.70 for the failed-yes/no and concomitant preventive-yes/no subgroups, respectively, confirming that the efficacy of erenumab 70 mg was similar regardless of whether a patient failed or did not fail a prior preventive treatment or was receiving or not receiving a concomitant treatment.

### Change from baseline in mean monthly acute MSM treatment days

**Fig. 3:**
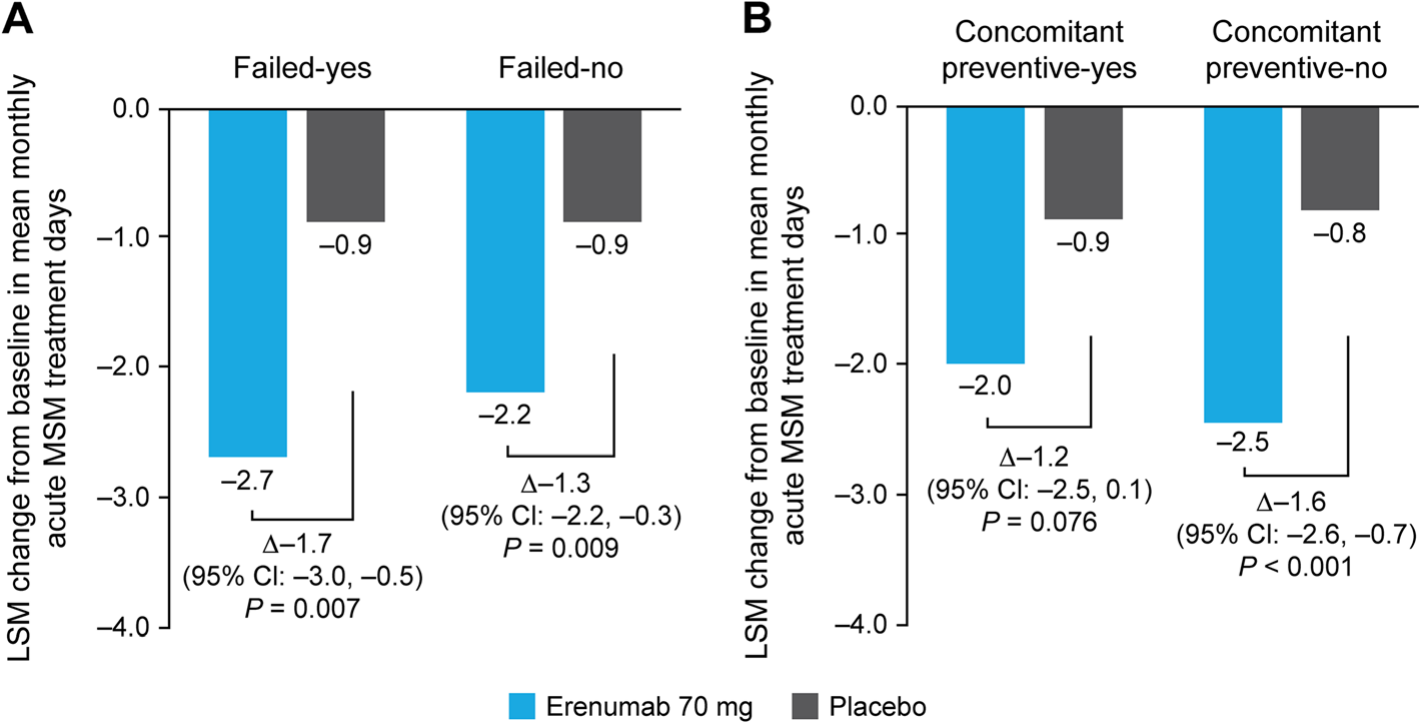
LSM change from baseline in mean monthly acute MSM treatment days in (**A**) failed-yes/no and (**B**) concomitant preventive-yes/no subgroups. *CI* confidence interval, *LSM* least squares mean, *MSM* migraine-specific medication

From baseline to Months 4–6, clinically relevant reductions in the mean number of monthly acute MSM treatment days were observed in patients receiving erenumab 70 mg versus placebo in all patient subgroups. The differences in LSM change from baseline in mean monthly acute MSM treatment days with erenumab 70 mg versus placebo were − 1.7 (95% CI: − 3.0, − 0.5; *P* = 0.007) and − 1.3 (95% CI: − 2.2, − 0.3; *P* = 0.009) in the failed-yes and failed-no subgroups, respectively. The differences in LSM change from baseline in mean monthly acute MSM treatment days with erenumab 70 mg versus placebo were − 1.2 (95% CI: − 2.5, 0.1; *P* = 0.076) and − 1.6 (95% CI: − 2.6, − 0.7; *P* < 0.001) in the concomitant preventive-yes and concomitant preventive-no subgroups, respectively (Fig. 3).

*P*-values for treatment-by-subgroup interactions were 0.57 and 0.55 for the failed-yes/no and concomitant preventive-yes/no subgroups, respectively, confirming that the efficacy of erenumab 70 mg was similar regardless of whether a patient failed or did not fail a prior preventive treatment or was receiving or not receiving a concomitant treatment.

### Tolerability

Overall, 60.3%–69.5% and 53.8%–65.0% of patients in the failed-yes and concomitant preventive-yes subgroups, respectively, had ≥1 TEAE. Across all subgroups, most TEAEs were CTCAE grade 2. The incidence of TEAEs for placebo compared with erenumab 70 mg was broadly comparable within each subgroup; however, a lower proportion of patients experienced ≥1 TEAE with placebo versus erenumab 70 mg in the concomitant preventive-yes subgroup (53.8% versus 65.0% of patients). The incidence of TEAEs in the concomitant preventive-yes subgroup treated with erenumab 70 mg was similar to the incidence in the concomitant preventive-no subgroup receiving erenumab 70 mg or placebo. The number of serious adverse events was low, and there were no adverse events leading to discontinuation of investigational product or fatal adverse events (Table 2).

**Table 2:** Summary of adverse events

N (%)	Patient subgroup							
	Prior preventive treatment failure				Concomitant preventive			
	Yes (*N* = 117)		No (*N* = 144)		Yes (*N* = 92)		No (*N* = 169)	
	Erenumab 70 mg (*N* = 59)	Placebo (*N* = 58)	Erenumab 70 mg (*N* = 71)	Placebo (*N* = 73)	Erenumab 70 mg (*N* = 40)	Placebo (*N* = 52)	Erenumab 70 mg (*N* = 90)	Placebo (*N* = 79)
≥1 TEAE	41 (69.5)	35 (60.3)	44 (62.0)	42 (57.5)	26 (65.0)	28 (53.8)	59 (65.6)	49 (62.0)
Grade ≥2	34 (57.6)	28 (48.3)	38 (53.5)	38 (52.1)	24 (60.0)	24 (46.2)	48 (53.3)	42 (53.2)
Grade ≥3	3 (5.1)	1 (1.7)	1 (1.4)	1 (1.4)	2 (5.0)	0 (0.0)	2 (2.2)	2 (2.5)
Grade ≥4	0 (0.0)	0 (0.0)	0 (0.0)	1 (1.4)	0 (0.0)	0 (0.0)	0 (0.0)	1 (1.3)
Any SAE	1 (1.7)	1 (1.7)	1 (1.4)	1 (1.4)	0 (0.0)	0 (0.0)	2 (2.2)	2 (2.5)
AE leading to discontinuation of investigational product	0 (0.0)	0 (0.0)	0 (0.0)	0 (0.0)	0 (0.0)	0 (0.0)	0 (0.0)	0 (0.0)
Fatal AE	0 (0.0)	0 (0.0)	0 (0.0)	0 (0.0)	0 (0.0)	0 (0.0)	0 (0.0)	0 (0.0)

Grading categories determined using Common Terminology Criteria for Adverse Events version 4.03. *AE* adverse event, *SAE* serious adverse event, *TEAE* treatment-emergent adverse event

## Discussion

The current subgroup analyses are the first to report efficacy and safety of erenumab 70 mg stratified by prior preventive treatment failure status in Japanese patients with migraine. Additionally, these are the first analyses to demonstrate efficacy of erenumab 70 mg in combination with concomitant preventive treatment. Erenumab 70 mg was associated with clinically relevant improvements across all efficacy endpoints versus placebo. Treatment effects were not significantly modified by prior and concomitant preventive treatment status. Overall, the current findings support global Phase 2 and 3 erenumab studies conducted outside of Japan [10–12], along with the recent Phase 2 study in Japan where erenumab 70 mg demonstrated statistically significant efficacy in patients with EM [14].

As clinically expected, the failed-yes subgroup had more severe disease than the failed-no subgroup, as reflected by the greater proportion of patients with CM and mean number of MMD and monthly acute MSM treatment days at baseline. Similarly, the concomitant preventive-yes subgroup had more severe disease than the concomitant preventive-no subgroup, reflected by the greater proportion of patients with prior treatment failure and CM. Notably, we observed the numerically largest reductions in mean MMD (both absolute and placebo-adjusted) in erenumab-treated patients in the failed-yes subgroup. This finding supports studies that report that erenumab is efficacious in patients with prior preventive treatment failure [15–17], and indicates that erenumab 70 mg is efficacious even in Japanese patients with severe disease. Patients with no therapeutic response to ≥3 prior migraine preventive classes were excluded from the current study; however, erenumab has been shown to be efficacious in difficult-to-treat patients with EM in whom 2–4 prior preventive treatments were unsuccessful [17].

With regard to tolerability, the safety profile of erenumab 70 mg was similar across subgroups and there were no new safety issues based on the established safety profile of erenumab 70 mg [13]. The majority of existing preventive treatments for migraine have undesirable side effects, including weight gain and dizziness [5–9], with a high risk of clinically significant drug-drug interactions [20]. These factors can contribute to failed adherence and reduced compliance [21–23]. Importantly, in the current study, the benefits of erenumab 70 mg in combination with concomitant preventive treatment were not associated with an increased incidence of TEAEs relative to erenumab 70 mg without concomitant preventives or placebo without concomitant preventives. The similarity in tolerability between erenumab and placebo in difficult-to-treat migraine patients has also been previously demonstrated in CM patients with medication overuse [24].

The current study had limitations. The study was not powered to demonstrate efficacy in the patient subgroups and the efficacy analyses in the concomitant preventive treatment subgroups were post hoc; therefore, the results should be considered in the context of other evidence relating to erenumab. Additionally, there was a slight imbalance in the sample sizes of the patient subgroups, although this is not expected to significantly affect the results. Lastly, the current study reports efficacy and safety data from the 24-week DBTP only, thus longer-term erenumab efficacy and safety data in Japanese patients will be of interest.

## Conclusion

In conclusion, erenumab 70 mg was well tolerated and effective in Japanese patients who had either failed prior preventive treatment or had not failed, and in patients receiving erenumab 70 mg as monotherapy or in combination with another preventive agent.

## Supplementary Information

**Additional file 1:** **Supplemental Figure 1.** Study design.

**Additional file 2:** **Supplemental Table 1.** Categories of concomitant preventive treatments.

## Data Availability

Qualified researchers may request data from Amgen clinical studies. Complete details are available at www.amgen.com/datasharing.

## Abbreviations

CI: Confidence interval
CM: Chronic migraine
CMH: Cochran-Mantel-Haenszel test
CTCAE: Common Terminology Criteria for Adverse Events
DBTP: Double-blind treatment phase
eDiary: Electronic diary
EM: Episodic migraine
LSM: Least squares mean
MMD: Monthly migraine days
MSM: Migraine-specific medication
OR: Odds ratio
SD: Standard deviation
TEAE: Treatment-emergent adverse event
