# Supplementary material for: Efficacy and safety of erenumab in Japanese migraine patients with prior preventive treatment failure or concomitant preventive treatment: subgroup analyses of a phase 3, randomized trial
Source: J Headache Pain. 2021 Sep 18;22(1):110. doi: 10.1186/s10194-021-01313-8 (PMC8449906; doi:10.1186/s10194-021-01313-8)
Supplement: Supplementary file 1 — Additional file 1: Supplemental Figure 1. Study design. [file 10194_2021_1313_MOESM1_ESM.docx]

**Additional File 1**

Supplemental Figure 1. Study design.


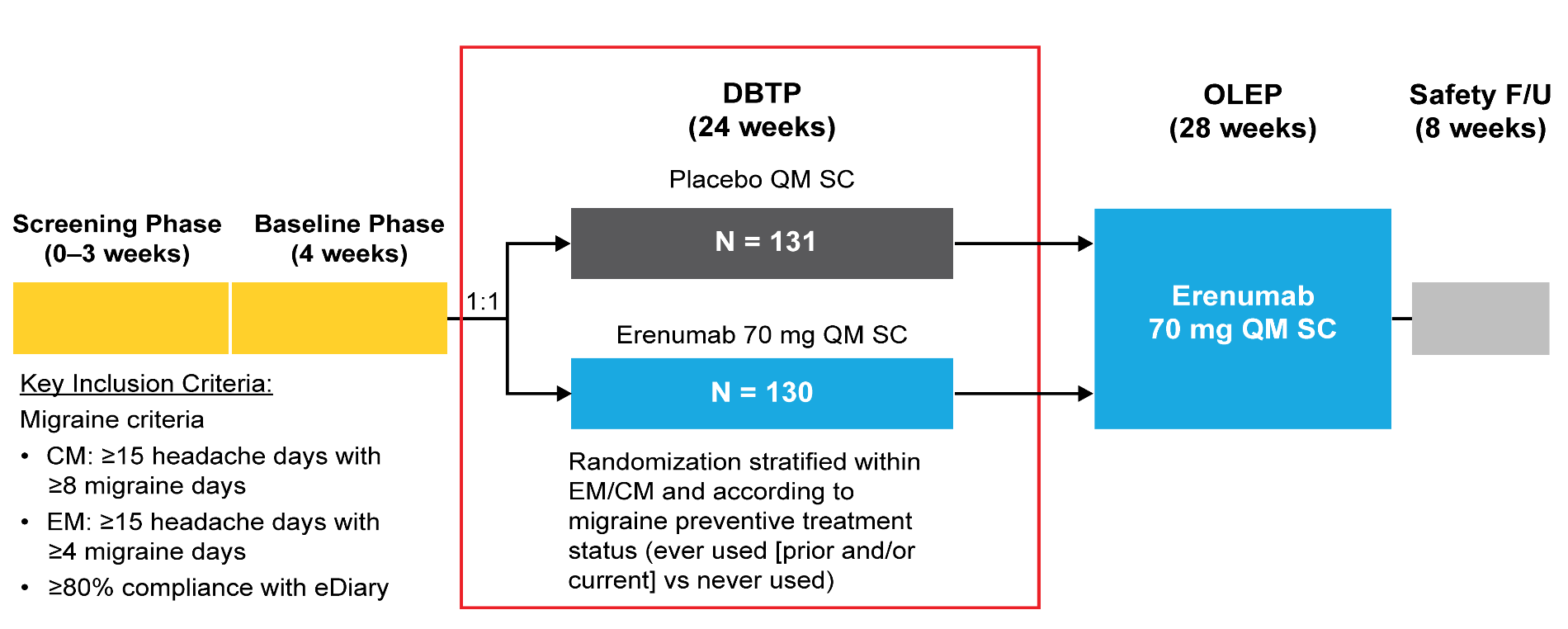


Efficacy data from the 24-week DBTP were used for these analyses. CM, chronic migraine; DBTP, double-blind treatment phase; eDiary, electronic diary; EM, episodic migraine; F/U, follow-up; OLEP, open-label extension period; QM, once monthly; SC, subcutaneous.
