# Supplementary material for: Efficacy and safety of erenumab in Japanese migraine patients with prior preventive treatment failure or concomitant preventive treatment: subgroup analyses of a phase 3, randomized trial
Source: J Headache Pain. 2021 Sep 18;22(1):110. doi: 10.1186/s10194-021-01313-8 (PMC8449906; doi:10.1186/s10194-021-01313-8)
Supplement: Supplementary file 2 — Additional file 2: Supplemental Table 1. Categories of concomitant preventive treatments. [file 10194_2021_1313_MOESM2_ESM.docx]

**Additional File 2**

Supplemental Table 1. Categories of concomitant preventive treatments.

| Treatment, n (%) | Concomitant preventive-yes (N=92)^a^ | |
| --- | --- | --- |
|  | Erenumab 70 mg (N=40) | Placebo (N=52) |
| Divalproex/valproate | 8 (20.0) | 10 (19.2) |
| Topiramate | 3 (7.5) | 0 (0.0) |
| Beta-blockers | 7 (17.5) | 4 (7.7) |
| Tricyclic antidepressants | 9 (22.5) | 12 (23.1) |
| Serotonin-norepinephrine reuptake inhibitors | 0 (0.0) | 2 (3.8) |
| Flunarizine/verapamil/lomerizine | 7 (17.5) | 15 (28.8) |
| Lisinopril/candesartan | 1 (2.5) | 1 (1.9) |
| Botulinum toxin | 0 (0.0) | 0 (0.0) |
| Other^b^ | 7 (17.5) | 14 (26.9) |

^a^Patients may have contributed to more than one category.

^b^Other may include butterbur/feverfew/magnesium (≥600 mg/day), riboflavin (≥100 mg/day), clonidine/guanfacine, cyproheptadine, methysergide, pizotifen, carbamazepine, and gabapentin.
